# The achievement of comprehensive control targets among type 2 diabetes mellitus patients of different ages

**DOI:** 10.18632/aging.103358

**Published:** 2020-07-22

**Authors:** Yun Yu, Kaipeng Xie, Qinglin Lou, Hui Xia, Dan Wu, Lingli Dai, Cuining Hu, Kunlin Wang, Shan Shan, Yun Hu, Wei Tang

**Affiliations:** 1 Department of Endocrinology and Metabolism, Geriatric Hospital of Nanjing Medical University, Nanjing, China; 2 Nanjing Maternity and Child Health Care Institute, Nanjing Maternity and Child Health Care Hospital, Women's Hospital of Nanjing Medical University, Nanjing, China; 3 Division of Geriatrics, Drum Tower Clinical Medical College of Nanjing Medical University, Nanjing, China

**Keywords:** age, type 2 diabetes mellitus, comprehensive target control, achievement

## Abstract

Objective: To evaluate achievement of comprehensive controls among patients with type 2 diabetes mellitus (T2DM) in different age groups.

Results: The elderly patients had higher control rates for BMI (44.36%), TC (50.83%) and LDL-C (48.27%) than those aged 60-80 years and younger patients (all *P* <0.05). Multiple logistic regression revealed that elderly patients were more likely to achieve control targets for HbA1c (odd ratio (OR) = 2.19), TC (OR = 1.32), HDL-C (OR = 1.35), and TG (OR = 1.74) than younger patients. This effect was stronger in males (OR_HbA1c_ = 2.27; OR_TC_ = 1.41; OR_HDL-C_ = 1.51; OR_TG_ = 1.80). By contrast, elderly females were only more likely to achieve HbA1c < 7.0% (OR=1.88).

Conclusions: Our findings suggest that comprehensive control strategies still should be strengthened.

Methods: A total of 3126 T2DM patients were included, and detected blood pressure (BP), body mass index (BMI), glycosylated hemoglobin (HbA1c), fasting plasma glucose (FPG), postprandial plasma glucose (PPG), total cholesterol (TC), low density lipoprotein cholesterol (LDL-C), triglycerides (TG), and high-density lipoprotein cholesterol (HDL-C). We divided patients into three age groups (<60, 60-80 and ≥ 80 years), to assess the differences in achieving the control targets.

## INTRODUCTION

Diabetes mellitus is an epidemic in China and globally [1, 2]. A large epidemiological survey showed that the prevalence of diabetes among adults in China was 11.6% [3]. The rising prevalence of diabetes also brings a heavy burden to medical care and clinical practice. Type 2 diabetes mellitus (T2DM) is the most common form, accounting for nearly 90% of all cases of diabetes [4]. In addition to the physical damage caused by hyperglycemia and diabetes-related complications, the unhealthful effects of T2DM are mainly due to comorbidities such as hypertension, cardiovascular disease (CVD), obesity, and dyslipidemia, which can lead to poor life quality and a shortened life expectancy [5].

According to the 2017 edition of China's guidelines for the prevention and treatment of T2DM, comprehensive management of T2DM involves the control of glycosylated hemoglobin (HbA1c < 7%) and other risk factors such as dyslipidemia, hypertension, body mass index (BMI), and the maintenance of plasma glucose levels within the target range [6]. Several previous studies have shown that good comprehensive management of T2DM is beneficial in reducing diabetic complications and comorbidities and necessary for both medicine and social economy [7, 8]. Unfortunately, a high proportion of patients with T2DM still fail to meet the comprehensive targets required in the guidelines in China, which may lead to severe diabetes-related complications and mortality [5, 9].

Since aging is a known risk factor for diabetes and because the elderly population has increased sharply in China and globally [10], it is of importance to consider age differences in T2DM management. However, studies exploring the differences in comprehensive targeted control of T2DM among patients of various ages are scarce. Therefore, this study aimed to assess the comprehensive control status of different aged patients with T2DM in China.

## RESULTS

### Baseline characteristics of patients with T2DM

Among the 3126 patients with T2DM included in our study, the median patient age was 68 (59-78) years old. There were 2124 (67.95%) male patients and 1002 (32.05%) female patients, with a median age of 66 (57-76) years in males and 71 (63-80) years in females. The characteristics of the included population according to sex are summarized in Table 1. Overall, male patients had a high median BMI (25.08 *vs* 24.53 kg/m^2^), FPG (7.10 *vs* 6.93 mmol/L) and HbA1c (7.35% *vs* 7.20%) and a lower median diabetic duration (2.83 *vs* 4.75 years), TC (4.56 *vs* 4.75 mmol/L), LDL-C (2.74 *vs* 2.79 mmol/L) and HDL-C (1.04 *vs* 1.16 mmol/L) than female patients (*P* < 0.05 for all comparisons). A greater proportion of female patients than male patients had hypertension (64.57% *vs* 57.77%, *P* <0.001) and used antihypertensive agents (53.69% *vs* 49.62%, *P* = 0.037).

**Table 1:** Baseline characteristics of the included patients with T2DM.

**Characteristic**	**Total**	**Male**	**Female**	***P* value**
Number of patients	3126	2124 (67.95)	1002 (32.05)	-
Age	68 (59-78)	66 (57-76)	71 (63-80)	<0.001
Diabetic duration (years)	3.33 (0.17-10.33)	2.83 (0.17-10.00)	4.75 (0.17-11.42)	<0.001
<5 [n (%)]	1759 (56.27%)	1256 (59.13)	503 (50.20)	<0.001
5-10 [n (%)]	514 (16.44%)	335 (15.78)	179 (17.86)	
≥10 [n (%)]	853 (27.29%)	533 (25.09)	320 (31.94)	
Hypertension [n (%)]				
Yes	1874 (59.95)	1227 (57.77)	647 (64.57)	<0.001
No	1252 (40.05)	897 (42.23)	355 (35.43)	
SDP (mmHg)	130 (120-140)	130 (120-140)	130 (120-140)	0.553
DBP (mmHg)	80 (70-82)	80 (70-84)	75 (70-80)	<0.001
BMI (kg/m^2^)	24.90 (22.95-27.14)	25.08 (23.18-27.17)	24.53 (22.41-27.11)	<0.001
FPG (mmol/L)	7.05 (6.10-8.50)	7.10 (6.10-8.60)	6.93 (5.95-8.20)	<0.001
PPG (mmol/L)	11.50 (9.00-15.50)	11.70 (9.00-15.80)	11.25 (8.90-15.10)	0.086
HbA1c (%)	7.3 (6.4-9.2)	7.35 (6.40-9.30)	7.20 (6.40-8.90)	0.042
TG (mmol/L)	1.43 (1.00-2.06)	1.45 (1.01-2.10)	1.39 (0.99-1.99)	0.068
TC (mmol/L)	4.64 (3.99-5.32)	4.56 (3.94-5.23)	4.75 (4.13-5.53)	<0.001
LDL-C (mmol/L)	2.76 (2.22-3.32)	2.74 (2.20-3.30)	2.79 (2.25-3.35)	0.034
HDL-C (mmol/L)	1.08 (0.91-1.30)	1.04 (0.89-1.24)	1.16 (0.98-1.40)	<0.001
Treatment [n (%)]				0.004
Diet and exercise alone	545 (17.43)	368 (17.33)	177 (17.67)	
1 OADs alone	1003 (32.09)	713 (33.57)	290 (28.94)	
2 OADs alone	655 (20.95)	456 (21.47)	199 (19.86)	
≥3 OADs alone	147 (4.70)	88 (4.14)	59 (5.89)	
Insulin alone	98 (3.14)	70 (3.30)	28 (2.79)	
Insulin+OADs	678 (21.69)	429 (20.20)	249 (24.85)	
Antihypertensive agents			0.037
Yes	1592 (50.93)	1054 (49.62)	538 (53.69)	
No	1534 (49.07)	1070 (50.38)	464 (46.31)	
ARBs or ACEIs				0.499
Yes	1055 (33.75)	708 (33.33)	347 (34.63)	
No	2071 (66.25)	1416 (66.67)	655 (65.37)	
Lipid-lowering agents				0.085
Yes	1395 (44.63)	925 (43.55)	470 (46.91)	
No	1731 (55.37)	1199 (56.45)	532 (53.09)	
Antiplatelet agents				0.964
Yes	1582 (50.61)	1076 (50.66)	506 (50.50)	
No	1544 (49.39)	1048 (49.34)	496 (49.50)	

Data are expressed as the median (Q1-Q3) or numbers and percentages, n (%); *P* values for comparison between males and females.

The characteristics of patients with T2DM according to age groups are described in Table 2. The patients included 801 younger patients, 1660 patients aged 60-80 years and 665 elderly patients. Notably, there were statistically significant increasing trends in the diabetic duration, proportion of patients with hypertension, and proportion of patients who used antihypertensive agent and ARBs or ACEIs among the different age groups; furthermore, there were decreasing trends in BMI, FPG, HbA1c, TG, TC, and LDL-C (all *P* < 0.05). We further analyzed the data among different age categories according to sex and observed discrepancies between males and females (Supplementary Tables 1 and 2).

**Table 2:** Characteristics of the included patients with T2DM in different age groups.

**Characteristic**	**Age (years)**			***P* value**
	**<60**	**60-80**	**≥80**	
Number of patients	801	1660	665	
Diabetic duration(years)	0.42 (0.08-4.92)^a^	3.79 (0.17-10.42)^b^	8.17 (1.42-15.75) ^c^	<0.001
<5 [n (%)]	602 (75.16)^a^	897 (54.04)^b^	260 (39.10)^c^	<0.001
5-10 [n (%)]	115 (14.36)^a^	295 (17.77)^a^	104 (15.64)^a^	
≥10 [n (%)]	84 (10.49)^a^	468 (28.19)^b^	301 (45.26)^c^	
Hypertension [n (%)]				<0.001
Yes	308 (38.45)^a^	1051 (63.31)^b^	515 (77.44)^c^	
No	493 (61.55)	609 (36.69)	150 (22.56)	
SDP (mmHg)	125 (120-135)^a^	130 (120-140)^b^	130 (120-140)^c^	<0.001
DBP (mmHg)	80 (71-88)^a^	80 (70-82)^b^	70 (69-80)^c^	<0.001
BMI (kg/m^2^)	25.40 (23.34-27.68)^a^	24.91 (23.03-27.11)^b^	24.34 (22.31-26.67)^c^	<0.001
FPG (mmol/L)	7.60 (6.50-9.60)^a^	7.00 (6.00-8.23)^b^	6.70 (5.80-7.90)^c^	<0.001
PPG (mmol/L)	12.70 (9.70-17.0)^a^	11.10 (8.90-15.10)^b^	11.10 (8.70-14.83)^b^	<0.001
HbA1c (%)	8.10 (6.8-10.20)^a^	7.15 (6.40-9.00)^b^	7.00 (6.40-8.20)^b^	<0.001
TG (mmol/L)	1.67 (1.14-2.50)^a^	1.38 (0.99-1.98)^b^	1.30 (0.93-1.80) ^c^	<0.001
TC (mmol/L)	4.78 (4.17-5.43)^a^	4.62 (3.99-5.29)^b^	4.48 (3.83-5.20)^c^	<0.001
LDL-C (mmol/L)	2.81 (2.32-3.36)^a^	2.77 (2.22-3.33)^a^	2.64 (2.07-3.22)^c^	<0.001
HDL-C (mmol/L)	1.02 (0.87-1.20)^a^	1.10 (0.93-1.32)^b^	1.11 (0.95-1.34)^b^	<0.001
Treatment [n (%)]				<0.001
Diet and exercise alone	146 (18.23)^a^	298 (17.95)^a^	101 (15.19)^a^	
1 OADs alone	289 (36.08)^a^	519 (31.27)^a,b^	195 (29.32)^b^	
2 OADs alone	175 (21.85)^a^	344 (20.72)^a^	136 (20.45)^a^	
≥3 OADs alone	18 (2.25)^a^	100 (6.02)^b^	29 (4.36)^a,b^	
Insulin alone	19 (2.37)^a^	51 (3.07)^a^	28 (4.21)^a^	
Insulin+OADs	154 (19.23)^a^	348 (20.96)^a^	176 (26.47)^b^	
Antihypertensive agents				<0.001
Yes	254 (31.71)^a^	877 (52.83)^b^	461 (69.32)^c^	
No	547 (68.29)	783 (47.17)	204 (30.68)	
ARBs or ACEIs				<0.001
Yes	192 (23.97)^a^	566 (34.10)^b^	297 (44.66)^c^	
No	609 (76.03)	1094 (65.90)	368 (55.34)	
Lipid-lowering agents				0.096
Yes	349 (43.57)^a^	769 (46.33)^a^	277 (41.65)^a^	
No	452 (56.43)	891 (53.67)	388 (58.35)	
Antiplatelet agents				<0.001
Yes	338 (42.20)^a^	887 (53.43)^b^	357 (53.68)^b^	
No	463 (57.80)	773 (46.57)	308 (46.32)	

Data are expressed as the median (Q1-Q3) or numbers and percentages, n (%); *P* values for comparison over all 3 categories.

^a, b, c^ Each different subscript letter denotes a subset of age categories whose column proportions differ significantly from each other at *P* < 0.05.

### Control rates of metabolic targets in patients with T2DM according to different age groups

Control rates of metabolic targets in patients with T2DM stratified by different age groups are shown in Table 3. Most patients (62.51%) achieved a TG level < 1.7 mmol/L, nearly half of patients (48.91%) achieved the target for HDL-C and less than half of the patients achieved the other targets (BP, 30.84%; BMI, 37.75%; HbA1c, 41.71%; TC, 44.40%; LDL-C, 42.64%; triple metabolic control, 6.53%). Significant differences in the achieved control rates for each metabolic index presented above were observed across various age groups (all *P* < 0.001). No significant difference in the control rate for BP was observed across various age groups (*P* = 0.421). Notably, the control rates for the targets for BMI, TG, TC, and LDL-C were significantly the highest in elderly patients compared with the other two age groups (all *P* < 0.05). Patients aged from 60-80 years and elderly patients had significantly higher control rates for HbA1c, TG and triple metabolic control than younger patients (all *P* < 0.05). Furthermore, we analyzed the data among different age categories according to sex and found that the abovementioned significant difference that was present across different age groups remained in males but not in females (Supplementary Table 3).

**Table 3:** Control rates of targets among patients with T2DM by different age groups.

**Characteristic**	**Total**	**Age (years)**			***P* value**
		**<60**	**60-80**	**≥80**	
Number	3126	801	1660	665	
Blood pressure (mmHg)				0.421
<130/80	964 (30.84)	248 (30.96)	498 (30.00)	218 (32.78)	
≥130/80	2162 (69.16)	553 (69.04)	1162 (70.00)	447 (67.22)	
BMI (kg/m^2^)					<0.001
<24	1180 (37.75)	270 (33.71) ^a^	615 (37.05) ^a^	295 (44.36) ^b^	
24-28	1367 (43.73)	354 (44.19) ^a^	745 (44.88) ^a^	268 (40.30) ^a^	
≥28	579 (18.52)	177 (22.10) ^a^	300 (18.07) ^a,b^	102 (15.34) ^b^	
HbA1c (%)					<0.001
<7	1304 (41.71)	233 (29.09) ^a^	745 (44.88) ^b^	326 (49.02) ^b^	
≥7	1822 (58.29)	568 (70.91)	915 (55.12)	339 (50.98)	
TG (mmol/L)					<0.001
<1.7	1954 (62.51)	404 (50.44) ^a^	1083 (65.24) ^b^	467 (70.23) ^b^	
≥1.7	1172 (37.49)	397 (49.56)	577 (34.76)	198 (29.77)	
TC (mmol/L)					<0.001
<4.5	1388 (44.40)	305 (38.08) ^a^	745 (44.88) ^b^	338 (50.83) ^c^	
≥4.5	1738 (55.60)	496 (61.92)	915 (55.12)	327 (49.17)	
LDL-C (mmol/L)					
<2.6	1333 (42.64)	310 (38.70) ^a^	702 (42.29) ^a^	321 (48.27) ^b^	0.001
≥2.6	1793 (57.36)	491 (61.30)	958 (57.71)	344 (51.73)	
HDL-C					
Achieved target*	1529 (48.91)	354 (44.19) ^a^	851 (51.27) ^b^	324 (48.72) ^a,b^	0.004
Did not achieve target*	1597 (51.09)	447 (55.81)	809 (48.73)	341 (51.28)	
Triple metabolic control rate				0.016
Yes	204 (6.53)	35 (4.37) ^a^	120 (7.23) ^b^	49 (7.37) ^b^	
No	2922 (93.47)	766 (95.63)	1540 (92.77)	616 (92.63)	

Data are expressed as numbers and percentages, n (%); *P* values for comparison over all 3 categories.

*Achieved target means the levels of HDL-C >1.0 mmol/L in males or >1.3 mmol/L in females; Did not achieve target means the levels of HDL-C ≤1.0 mmol/L in males or ≤1.3 mmol/L in females.

^a, b, c^ Each different subscript letter denotes a subset of age categories whose column proportions differ significantly from each other at *P* < 0.05.

### Differences in achieving control targets among patients with T2DM by different age groups

In the logistic regression models without adjustment (model 1), with adjustment for sex, diabetic duration and BMI (model 2) and plus hypertension, FPG and PPG (model 3), positive ORs of achieved control targets for HbA1c, HDL-C and TG were observed in patients aged 60-80 years and elderly patients across different models (Table 4). In model 3, patients aged 60-80 years had 79% (OR = 1.79, 95% CI =1.40-2.30), 48% (OR = 1.48, 95% CI = 1.22-1.78) and 64% (OR = 1.64, 95% CI = 1.36-1.97) significantly increased odds of achieving control targets for HbA1c, HDL-C and TG, respectively. A stronger association between elderly age and achieving the control targets for HbA1c (OR = 2.19, 95% CI = 1.60-2.99), TG (OR = 1.74, 95% CI = 1.36-2.23) and TC (OR = 1.32, 95% CI = 1.05-1.67) was observed.

**Table 4:** Odds ratios (95% CI) for achieved comprehensive control targets by different age groups among 3126 patients with T2DM (Ref.<60 years).

**Characteristic**	**Age (years)**	
	**60~80**	**≥80**
HbA1c < 7%		
Model 1	**1.98 (1.66,2.38)**	**2.34 (1.89,2.91)**
Model 2	**2.34 (1.89,2.91)**	**3.31 (2.62,4.18)**
Model 3	**1.79 (1.40,2.30)**	**2.19 (1.60,2.99)**
BP < 130/80 mmHg		
Model 1	0.96 (0.80,1.15)	1.09 (0.87,1.36)
Model 2	0.89 (0.74,1.08)	0.95 (0.75,1.21)
Model 3	1.01 (0.83,1.23)	1.18 (0.92,1.52)
TC < 4.5 mmol/L		
Model 1	**1.32 (1.11,1.57)**	**1.68 (1.36,2.07)**
Model 2	**1.29 (1.08,1.53)**	**1.53 (1.22,1.91)**
Model 3	1.18 (0.98,1.42)	**1.32 (1.05,1.67)**
LDL-C < 2.6 mmol/L		
Model 1	1.16 (0.98,1.38)	**1.48 (1.20,1.82)**
Model 2	1.09 (0.91,1.30)	**1.27 (1.02,1.59)**
Model 3	1.00 (0.83,1.20)	1.11 (0.88,1.41)
Achieved HDL-C target		
Model 1	**1.33 (1.12,1.57)**	**1.20 (0.98,1.47)**
Model 2	**1.53 (1.28,1.83)**	**1.42 (1.13,1.78)**
Model 3	**1.48 (1.22,1.78)**	**1.35 (1.06,1.72)**
TG < 1.7 mmol/L		
Model 1	**1.84 (1.55,2.19)**	**2.32 (1.87,2.88)**
Model 2	**1.68 (1.40,2.01)**	**1.82 (1.44,2.30)**
Model 3	**1.64 (1.36,1.97)**	**1.74 (1.36,2.23)**

Model 1: unadjusted model.

Model 2: adjusted for sex, diabetic duration (years), and BMI (kg/m^2^).

Model 3: adjusted for sex, diabetic duration (years), BMI (kg/m^2^), hypertension (yes or no), FPG (mmol/L), and PPG (mmol/L).

We further analyzed the data among different age categories according to sex (Table 5). Multiple logistic regression revealed that compared with younger patients, male patients aged 60-80 years were significantly associated with achieving targets for HbA1c (OR = 1.83, 95% CI = 1.37-2.45), TC (OR = 1.28, 95% CI = 1.04-1.58), HDL-C (OR = 1.47, 95% CI = 1.19-1.82), and TG (OR = 1.67, 95% CI = 1.35-2.08). Furthermore, elderly male patients were more likely to achieve the targets for HbA1c (OR = 2.27, 95% CI = 1.54-3.35), BP (OR = 1.40, 95% CI = 1.02-1.93), TC (OR = 1.41, 95% CI = 1.06-1.88), HDL-C (OR = 1.51, 95% CI = 1.13-2.03), and TG (OR = 1.80, 95% CI = 1.32-2.44) than younger patients. However, we only observed that elderly females were more likely to achieve HbA1c (OR = 1.88, 95% CI = 1.07-3.31).

**Table 5:** Odds ratios (95% CI) for achieved comprehensive control targets by different age groups in male and female patients (Ref.<60).

**Characteristic**	**Male**		**Female**	
	**60-80**	**≥80**	**60-80**	**≥80**
HbA1c < 7%				
Model 1	**2.18 (1.76,2.69)**	**2.75 (2.11,3.58)**	1.38 (0.96,1.99)	1.48 (0.99,2.21)
Model 2	**2.49 (2.01,3.09)**	**3.72 (2.80,4.95)**	**1.85 (1.26,2.70)**	**2.46 (1.59,3.79)**
Model 3	**1.83 (1.37,2.45)**	**2.27 (1.54,3.35)**	1.55 (0.95,2.54)	**1.88 (1.07,3.31)**
BP < 130/80 mmHg				
Model 1	1.10 (0.88,1.37)	1.28 (0.97,1.69)	**0.49 (0.34,0.71)**	**0.52 (0.35,0.78)**
Model 2	1.05 (0.83,1.31)	1.13 (0.84,1.52)	**0.54 (0.37,0.78)**	**0.58 (0.38,0.88)**
Model 3	1.19 (0.94,1.51)	**1.40 (1.02,1.93)**	0.60 (0.41,0.88)	0.72 (0.46,1.12)
TC < 4.5 mmol/L				
Model 1	**1.51 (1.24,1.84)**	**1.93 (1.50,2.50)**	1.05 (0.72,1.51)	1.42 (0.95,2.12)
Model 2	**1.38 (1.13,1.69)**	**1.59 (1.21,2.08)**	0.97 (0.67,1.41)	1.22 (0.80,1.86)
Model 3	**1.28 (1.04,1.58)**	**1.41 (1.06,1.88)**	0.87 (0.59,1.28)	1.01 (0.65,1.56)
LDL-C < 2.6 mmol/L				
Model 1	1.11 (0.91,1.35)	**1.60 (1.24,2.07)**	1.38 (0.95,2.00)	1.45 (0.97,2.19)
Model 2	1.02 (0.84,1.25)	**1.35 (1.03,1.77)**	1.29 (0.89,1.89)	1.25 (0.82,1.92)
Model 3	0.97 (0.78,1.19)	1.24 (0.93,1.64)	1.14 (0.78,1.68)	1.01 (0.65,1.57)
Achieved HDL-C target				
Model 1	**1.58 (1.30,1.92)**	**1.74 (1.35,2.25)**	1.35 (0.92,1.99)	1.06 (0.69,1.63)
Model 2	**1.54 (1.25,1.88)**	**1.61 (1.23,2.13)**	1.44 (0.97,2.15)	1.08 (0.69,1.70)
Model 3	**1.47 (1.19,1.82)**	**1.51 (1.13,2.03)**	1.42 (0.94,2.13)	1.07 (0.67,1.72)
TG < 1.7 mmol/L				
Model 1	**2.09 (1.72,2.55)**	**2.89 (2.20,3.80)**	1.17 (0.81,1.68)	1.32 (0.88,1.98)
Model 2	**1.80 (1.46,2.21)**	**2.01 (1.50,2.69)**	1.19 (0.82,1.73)	1.27 (0.83,1.95)
Model 3	**1.67 (1.35,2.08)**	**1.80 (1.32,2.44)**	1.27 (0.87,1.87)	1.39 (0.89,2.18)

Model 1: unadjusted model

Model 2: adjusted for sex, diabetic duration (years), and BMI (kg/m^2^).

Model 3: adjusted for sex, diabetic duration (years), BMI (kg/m^2^), hypertension (yes or no), FPG (mmol/L), and PPG (mmol/L).

## DISCUSSION

In the present study, we assessed the comprehensive control of T2DM in patients according to different age groups. Two age groups (60-80 years and ≥ 80 years) were more likely to achieve the comprehensive control targets, including HbA1c < 7.0%, HDL-C, and TG < 1.7 mmol/L, than younger patients aged <60 years. In addition, the control target for TC was significantly associated with age ≥ 80 years. The strength of these associations was consistently stronger in male patients aged ≥ 80 years. Notably, the only association among female patients was the target for HbA1c in elderly patients aged ≥ 80 years.

HbA1c is usually used as an indicator of glycemic control. Most guidelines recommend HbA1c **<**7% as good glycemic control for most adults with T2DM [6, 11, 12]. Our study showed that only 41.71% of the patients achieved the target for HbA1c **<**7.0%, which was similar to the findings of two other Chinese studies (42.3% [13] and 39.7% [14]). The reasons for the failure to achieve the glycemic control target are multifactorial and may include poor adherence to medications [15] and a lack of knowledge regarding diabetic management and self-care [16]. Additionally, we found that the proportion of HbA1c < 7.0% in patients aged ≥ 80 years was higher than that in younger patients. This finding was consistent with the findings of another study [17]. Although the group of patients aged ≥ 80 years had a significantly longer diabetic duration and a higher proportion of patients received insulin treatment (with OADs) than the group of younger patients, these factors did not affect glycemic control. This result suggests that satisfactory management of elderly patients with diabetes is the most important determinant of glycemic control, and age is not an obstacle.

According to the Chinese guidelines for T2DM in 2017, the rates of achieving the control targets for TG, TC, LDL-C and HDL-C in our study were 62.51%, 44.40%, 42.64% and 48.91%, respectively. Additionally, the proportion of patients achieving the control targets for lipid profiles (TG, TC and LDL-C) was higher among patients aged ≥ 80 years than among younger patients aged <60 years old. We also found that 43.57% of the patients took lipid-lowering drugs. Studies have suggested that patients with T2DM with high LDL-C are less likely to receive appropriate lipid-lowering therapy [18, 19]. Furthermore, poor lipid control might be related to a lack of dietary restriction [18]. Therefore, lipid control is also important for diabetic management.

In our study, only approximately one-third of the patients with T2DM achieved a BP**<**130/80 mmHg (30.84%), which was close to the findings of another study (35.3%) [20], and the proportion of patients achieving the target for BP was similar among the different age groups (*P* = 0.421). Achieving BP control is beneficial to patients with T2DM for reducing CVD and mortality [21]. In our study, no significant associations between the achieved control target for BP and different age groups was observed in all patients. Therefore, BP control should be one of the main treatment priorities for these patients, and further attention is warranted.

Previous studies have reported that older patients with T2DM are more likely to achieve the control targets for glycemia and other CVD risk factors than younger patients [22, 23]. In the present study, logistic regression analysis showed that after multiple adjustments for confounders, patients aged ≥ 80 years were more likely to achieve the control targets for HbA1c, TC, HDL-C, and TG than younger patients aged <60 years. The reasons for this finding are still unclear. One explanation could be that older patients pay more attention to their diabetes and have better compliance with medication and self-management, including lifestyle control [23]. Furthermore, older people sometimes are under the care of family members or specialized centers. Another reason might be that patients aged ≥ 80 years are survivors with better comprehensive control of T2DM and have outlived their contemporaries who did not achieve integrative control. As a result, integrative management for elderly patients with T2DM could improve their quality of life and functional capability. Additionally, BMI is commonly used to identify overweight and obesity and is closely related to diabetes [24]. A previous study demonstrated that BMI was an important risk factor for glycemic control [5]. Our study also found that the proportion of patients with BMI < 24 kg/m^2^ was higher in patients aged ≥ 80 years than in younger patients, which may partly explain why glycemic control was better in these patients.

In this study, we further analyzed the differences in achieving comprehensive control targets among different age categories according to sex. We found that males with T2DM aged ≥80 years were more likely to achieve HbA1c, BP, TC, HDL-C, and TG targets than patients aged <60 years. However, females aged ≥80 years with T2DM were only more likely to achieve the HbA1c target. This finding may be attributed to the differences in sex hormone levels, body fat distribution, and compliance. Postmenopausal hypogonadism results in increasing total and central fat [25]. A previous study showed that long-term hormone therapy in postmenopausal women was associated with healthier body fat distribution and better adipocytokine profiles [26]. Females aged ≥80 years in our study were all postmenopausal women without hormone therapy; thus, we did not observe similar results with females as with males. The mechanisms underlying the differences between males and females are still unclear, and further studies are warranted to clarify this issue.

Our study had some limitations. The study collected no data about the level of education, compliance and care management knowledge of the patients, which may be useful for the analysis of the comprehensive control of T2DM. In addition, this study lacked relevant data on diabetic complications, which could make the results more comprehensive.

In conclusion, most patients with T2DM in Nanjing had unsatisfactory control of glycemia, BP, lipids, and BMI. Males aged ≥ 80 years with T2DM were more likely to achieve the HbA1c, BP, TC, HDL-C, and TG targets than younger patients (aged <60 years).

## MATERIALS AND METHODS

### Subjects

Between Jan 2013 and Dec 2018, 4697 patients with T2DM visited the Geriatric Hospital of Nanjing Medical University in Nanjing, China. The diagnosis of T2DM was made based on the World Health Organization 1999 diagnostic criteria. Namely, a fasting plasma glucose ≥7.0 mmol/L or a 2-h plasma glucose ≥11.1 mmol/L during an oral glucose tolerance test (OGTT) or a random plasma glucose ≥11.1 mmol/L with classic symptoms of hyperglycemia was defined as diabetes mellitus [27]. A total of 3126 patients with T2DM were finally eligible for analysis according to the following exclusion criteria: 1) age less than 18 years old, 2) type 1 diabetes or secondary diabetes, 3) gestational diabetes, 4) active cancer, autoimmune diseases or infectious disease, and 5) any missing data for blood pressure (BP), BMI, HbA1c, fasting plasma glucose (FPG), postprandial plasma glucose (PPG), and lipid profiles including total cholesterol (TC), low density lipoprotein cholesterol (LDL-C), triglycerides (TG), and high-density lipoprotein cholesterol (HDL-C). Patients were divided into three groups by age: <60 years (younger patients), 60-80 years, and ≥ 80 years (elderly patients). The study protocol was approved by the Ethics Committee of the Geriatric Hospital of Nanjing Medical University. Written informed consent was obtained from each patient.

### Anthropometric and biochemical measurements

All patients were examined by collecting their clinical data, including age, sex, diabetic duration, BP, weight, height and therapeutic regimen (use of antihyperglycemic agents (including oral antidiabetic drugs (OADs) and insulin), antihypertensive agents (including angiotensin receptor blockers (ARBs) or angiotensin-converting enzyme inhibitors (ACEIs)), lipid-lowering agents and antiplatelet agents)**.** Overnight fasting blood samples were obtained for biochemical determinations of related factors such as HbA1c, FPG, PPG, and lipid profiles. All blood samples were tested at the central laboratory of the Geriatric Hospital of Nanjing Medical University.

### Comprehensive control targets for T2DM

According to the Chinese guidelines for T2DM in 2017, comprehensive management of T2DM includes the following control targets: HbA1c < 7%; BP<130/80 mmHg; TG <1. 7 mmol/L; TC < 4. 5 mmol/L; LDL-C <2. 6 mmol/L; HDL-C>1.0 mmol/L in males or >1.3 mmol/L in females; and BMI<24. 0 kg/m^2^ [6]. In addition, we defined simultaneously achieving HbA1c < 7%, BP <130/80 mmHg and LDL-C < 2.6 mmol/L as a qualified triple metabolic control.

### Statistical analysis

Continuous variables with a non-normal distribution are expressed as the median (interquartile range), and categorical variables are expressed as numbers (proportions). The Mann–Whitney U test for continuous variables and the χ2 test for categorical variables were used to compare the differences in baseline characteristics between the male and female groups. Significant differences between different age groups were evaluated by the Kruskal-Wallis test, the χ2 test and Fisher’s exact test as appropriate and followed by multiple comparison tests. Logistic regressions were performed to calculate the unadjusted and adjusted odds ratios (ORs) and 95% confidence intervals (CIs) for the achieved T2DM control targets in different age groups. The adjusted confounders included sex, diabetic duration, BMI, hypertension, FPG, and PPG. Patients aged < 60 years were the reference group. The criterion for statistical significance was a *P*-value < 0.05. All calculations were performed using SPSS software version 20.0 (SPSS version 20.0; IBM Corporation, Armonk, NY, USA) and R version 3.5.

## Supplementary Material

Supplementary Tables 1 and 2

Supplementary Table 3
