# Supplementary material for: The achievement of comprehensive control targets among type 2 diabetes mellitus patients of different ages
Source: Aging (Albany NY). 2020 Jul 22;12(14):14066–79. doi: 10.18632/aging.103358 (PMC7425513; doi:10.18632/aging.103358)
Supplement: Supplementary Tables 1 and 2 [file aging-12-103358-s002..pdf]

SUPPLEMENTARY TABLES

Supplementary Table 1. Characteristics of male patients with T2DM in different age groups.

Characteristic	Age (years)			*P* value
	<60	60~80	≥80	
Number of patients	648	1089	387	
Diabetic duration (years)	0.33 (0.08-4.83)[a]	3.42 (0.17-10.17)[b]	8.17 (0.63-15.71)[c]	<0.001
<5 [n (%)]	491 (75.77)[a]	609 (55.92)[b]	156 (40.31)[c]	<0.001
5-10 [n (%)]	91 (14.04)[a]	187 (17.17)[a]	57 (14.73)[a]	
≥10 [n (%)]	66 (10.19)[a]	293 (26.91)[b]	174 (44.96)[c]	
Hypertension				<0.001
Yes	248 (38.27)[a]	683 (62.72)[b]	296 (76.49)[c]	
No	400 (61.73)[a]	406 (37.28)[b]	91 (23.51)[c]	
SDP (mmHg)	125.5 (120.0-136.0)[a]	130 (120-140)[b]	130 (120-140)[c]	<0.001
DBP (mmHg)	80 (74-90)[a]	80 (70-84)[b]	73 (70-80)[c]	<0.001
BMI (kg/m^2)	25.65 (23.59-27.76)[a]	25.00 (23.26-27.10)[b]	24.38 (22.34-26.38)[c]	<0.001
FPG (mmol/L)	7.70 (6.50-9.60)[a]	7.00 (6.1-8.3)[b]	6.70 (5.90-7.87)[c]	<0.001
PPG (mmol/L)	12.90 (9.90-17.22)[a]	11.10 (9.00-15.00)[b]	11.00 (8.60-14.95)[b]	<0.001
HbA1c (%)	8.3 (6.8-10.2)[a]	7.1 (6.3-8.9)[b]	6.9 (6.4-8.2)[b]	<0.001
TG (mmol/L)	1.74 (1.21-2.71)[a]	1.37 (0.98-1.96)[b]	1.28 (0.86-1.72)[c]	<0.001
TC (mmol/L)	4.77 (4.15-5.42)[a]	4.53 (3.91-5.17)[b]	4.38 (3.71-5.04)[c]	<0.001
LDL-C (mmol/L)	2.79 (2.29-3.36)[a]	2.75 (2.22-3.30)[a]	2.57 (1.99-3.15)[c]	<0.001
HDL-C (mmol/L)	0.99 (0.85-1.16)[a]	1.06 (0.90-1.25)[b]	1.09 (0.92-1.31)[b]	<0.001
Treatment [n (%)]				0.003
Diet and exercise alone	123(18.98)[a]	187(17.17)[a]	58(14.99)[a]	
1 OAD alone	233(35.96)[a]	358(32.87)[a]	122(31.52)[a]	
2 OADs alone	148(22.84)[a]	229(21.03)[a]	79(20.41)[a]	
≥3 OADs alone	13(2.01)[a]	58(5.33)[b]	17(4.39)[a,b]	
Insulin alone	16(2.47)[a]	33(3.03)[a,b]	21(5.43)[b]	
Insulin+OAD	115(17.75)[a]	224(20.57)[a]	90(23.26)[a]	
Antihypertensive agents use				<0.001
Yes	211(32.56)[a]	576(52.89)[b]	267(68.99)[c]	
No	437(67.44)	513(47.11)	120(31.01)	
ARB or ACEI use				<0.001
Yes	166(25.62)[a]	368(33.79)[b]	174(44.96)[c]	
No	482(74.38)	721(66.21)	213(55.04)	
Lipid-lowering agent use				0.021
Yes	292(45.06)[a]	489(44.9)[a]	144(37.21)[b]	
No	356(54.94)	600(55.1)	243(62.79)	
Antiplatelet use				<0.001
Yes	283(43.67)[a]	591(54.27)[b]	202(52.2)[b]	
No	365(56.33)	498(45.73)	185(47.8)	

Data are expressed as the median (Q1-Q3) or numbers and percentages, n (%); *P* values for comparison over all 3 categories. [a, b, c] Each different subscript letter denotes a subset of age categories whose column proportions differ significantly from each other at $P < 0.05$.

Supplementary Table 2. Characteristics of female patients with T2DM in different age groups.

Characteristic	Age (years)			*P* value
	<60	60~80	≥80	
Number of patients	153	571	278	
Diabetic duration (years)	1.08 (0.71-5.42)[a]	4.58 (0.17-11.08)[b]	8.25 (2.54-15.73)[c]	<0.001
<5 [n (%)]	111 (72.55)[a]	288 (50.44)[b]	104 (37.41)[c]	<0.001
5-10 [n (%)]	24 (15.69)[a]	108 (18.91)[a]	47 (16.91)[a]	
≥10 [n (%)]	18 (11.76)[a]	175 (30.65)[b]	127 (45.68)[c]	
Hypertension [n (%)]				<0.001
Yes	60 (39.22)[a]	368 (64.45)[b]	219 (78.78)[c]	
No	93 (60.78)	203 (35.55)	59 (21.22)	
SDP (mmHg)	120 (110-130)[a]	130 (120-140)[b]	130 (120-140)[b]	<0.001
DBP (mmHg)	75 (70-80)[a]	78 (70-80)[a]	70 (68-80)[b]	<0.001
BMI (kg/m^2)	24.39 (22.19-26.71)	24.65 (22.58-27.18)	24.23 (22.31-27.11)	0.328
FPG (mmol/L)	7.30 (6.20-8.90)[a]	6.99 (6.00-8.20)[b]	6.68 (5.70-7.90)[c]	<0.001
PPG (mmol/L)	11.90 (9.20-16.00)	11.20 (8.80-15.10)	11.20 (8.90-14.65)	0.250
HbA1c (%)	7.8 (6.3-10.1)	7.2 (6.4-9.0)	7.0 (6.5-8.1)	0.046
TG (mmol/L)	1.40 (0.97-2.09)	1.39 (1.00-2.00)	1.39 (1.02-1.83)	0.994
TC (mmol/L)	4.80 (4.22-5.50)	4.80 (4.16-5.62)	4.63 (4.00-5.37)	0.107
LDL-C (mmol/L)	2.84 (2.45-3.36)	2.80 (2.23-3.40)	2.71 (2.22-3.27)	0.078
HDL-C (mmol/L)	1.13 (0.97-1.37)	1.17 (1.00-1.41)	1.16 (0.98-1.38)	0.318
Treatment [n (%)]				
Diet and exercise alone	23 (15.03)	111 (19.44)	43 (15.47)	0.049
1 OADs alone	56 (36.60)	161 (28.20)	73 (26.26)	
2 OADs alone	27 (17.65)	115 (20.14)	57 (20.50)	
≥3 OADs alone	5 (3.27)	42 (7.36)	12 (4.32)	
Insulin alone	3 (1.96)	18 (3.15)	7 (2.52)	
Insulin + OADs	39 (25.49)	124 (21.72)	86 (30.94)	
Antihypertensive agents				<0.001
Yes	43 (28.1)[a]	301 (52.71)[b]	194 (69.78)[c]	
No	110 (71.9)	270 (47.29)	84 (30.22)	
ARBs or ACEIs				<0.001
Yes	26 (16.99)[a]	198 (34.68)[b]	123 (44.24)[c]	
No	127 (83.01)	373 (65.32)	155 (55.76)	
Lipid-lowering agents				0.032
Yes	57 (37.25)[a]	280 (49.04)[b]	133 (47.84)[a,b]	
No	96 (62.75)	291 (50.96)	145 (52.16)	
Antiplatelet agents				<0.001
Yes	55 (35.95)[a]	296 (51.84)[b]	155 (55.76)[b]	
No	98 (64.05)	275 (48.16)	123 (44.24)	

Data are expressed as the median (Q1-Q3) or numbers and percentages, n (%); *P* values for comparison over all 3 categories.
[a, b, c] Each different subscript letter denotes a subset of age categories whose column proportions differ significantly from each other at $P < 0.05$.
