# Supplementary material for: The achievement of comprehensive control targets among type 2 diabetes mellitus patients of different ages
Source: Aging (Albany NY). 2020 Jul 22;12(14):14066–79. doi: 10.18632/aging.103358 (PMC7425513; doi:10.18632/aging.103358)
Supplement: Supplementary Table 3 [file aging-12-103358-s001..docx]

| **Supplementary Table 3. Control rates of targets in male and female patients with T2DM by different age groups.** | | | | | | | | | | | |
| --- | --- | --- | --- | --- | --- | --- | --- | --- | --- | --- | --- |
| Characteristic | | Age groups in male patients | | | | *P* value | Age groups in female patients | | | | *P* value |
|  |  | Total | <60 | 60~80 | ≥80 |  | Total | <60 | 60~80 | ≥80 |  |
| Number | | 2124 | 648 | 1089 | 387 |  | 1002 | 153 | 571 | 278 |  |
| Blood pressure (mmHg) | | |  |  |  | 0.207 |  |  |  |  | <0.001 |
|  | <130/80 | 601 (28.30) | 171 (26.39) | 308 (28.28) | 122 (31.52) |  | 363 (36.23) | 77 (50.33) ^a^ | 190 (33.27) ^b^ | 96 (34.53) ^b^ |  |
|  | ≥130/80 | 1523 (71.70) | 477 (73.61) | 781 (71.72) | 265 (68.48) |  | 639 (63.77) | 76 (49.67) | 381 (66.73) | 182 (65.47) |  |
| BMI (kg/m^2^) | |  |  |  |  | <0.001 |  |  |  |  | 0.628 |
|  | < 24 | 746 (35.12) | 200 (30.86) ^a^ | 379 (34.80) ^a^ | 167 (43.15) ^b^ |  | 434 (43.31) | 70 (45.75) | 236 (41.33) | 128 (46.04) |  |
|  | 24-28 | 989 (46.56) | 296 (45.68) ^a^ | 524 (48.12) ^a^ | 169 (43.67) ^a^ |  | 378 (37.73) | 58 (37.91) | 221 (38.70) | 99 (35.61) |  |
|  | ≥ 28 | 389 (18.31) | 152 (23.46) ^a^ | 186 (17.08) ^b^ | 51 (13.18) ^b^ |  | 190 (18.96) | 25 (16.34) | 114 (19.96) | 51 (18.35) |  |
| HbA1c (%) | |  |  |  |  | <0.001 |  |  |  |  | 0.144 |
|  | <7 | 860 (40.49) | 176 (27.16) ^a^ | 488 (44.81) ^b^ | 196 (50.65) ^b^ |  | 444 (44.31) | 57 (37.25) | 257 (45.01) | 130 (46.76) |  |
|  | ≥7 | 1264 (59.51) | 472 (72.84) | 601 (55.19) | 191 (49.35) |  | 558 (55.69) | 96 (62.75) | 314 (54.99) | 148 (53.24) |  |
| TG (mmol/L) | |  |  |  |  | <0.001 |  |  |  |  | 0.408 |
|  | <1.7 | 1313 (61.82) | 312 (48.15) ^a^ | 719 (66.02) ^b^ | 282 (72.87) ^c^ |  | 641 (63.97) | 92 (60.13) | 364 (63.75) | 185 (66.55) |  |
|  | ≥1.7 | 811 (38.18) | 336 (51.85) | 370 (33.98) | 105 (27.13) |  | 361 (36.03) | 61 (39.87) | 207 (36.25) | 93 (33.45) |  |
| TC (mmol/L) | |  |  |  |  | <0.001 |  |  |  |  | 0.089 |
|  | <4.5 | 985 (46.37) | 248 (38.27) ^a^ | 526 (48.30) ^b^ | 211 (54.52) ^b^ |  | 403 (40.22) | 57 (37.25) | 219 (38.35) | 127 (45.68) |  |
|  | ≥4.5 | 1139 (53.63) | 400 (61.73) | 563 (51.70) | 176 (45.48) |  | 599 (59.78) | 96 (62.75) | 352 (61.65) | 151 (54.32) |  |
| LDL-C (mmol/L) | |  |  |  |  | <0.001 |  |  |  |  | 0.167 |
|  | <2.6 | 911 (42.89) | 256 (39.51) ^a^ | 457 (41.97) ^a^ | 198 (51.16) ^b^ |  | 422 (42.12) | 54 (35.29) | 245 (42.91) | 123 (44.24) |  |
|  | ≥2.6 | 1213 (57.11) | 392 (60.49) | 632 (58.03) | 189 (48.84) |  | 580 (57.88) | 99 (64.71) | 326 (57.09) | 155 (55.76) |  |
| HDL-C | |  |  |  |  | <0.001 |  |  |  |  | 0.143 |
|  | Achieved target* | 1186 (55.84) | 308 (47.53) ^a^ | 641 (58.86) ^b^ | 237 (61.24) ^b^ |  | 343 (34.23) | 46 (30.07) | 210 (36.78) | 87 (31.29) |  |
|  | Did not achieve target* | 938 (44.16) | 340 (52.47) | 448 (41.14) | 150 (38.76) |  | 659 (65.77) | 107 (69.93) | 361 (63.22) | 191 (68.71) |  |
| Triple metabolic control rate | | |  |  |  | <0.001 |  |  |  |  | 0.282 |
|  | Yes | 118 (5.56) | 17 (2.62) ^a^ | 76 (6.98) ^b^ | 25 (6.46) ^b^ |  | 86 (8.58) | 18 (11.76) | 44 (7.71) | 24 (8.63) |  |
|  | No | 2006 (94.44) | 631 (97.38) | 1013 (93.02) | 362 (93.54) |  | 916 (91.42) | 135 (88.24) | 527 (92.29) | 254 (91.37) |  |
| Data are expressed as numbers and percentages, n (%); *P* values for comparison over all 3 categories.  *Achieved target means the levels of HDL-C >1.0 mmol/L in males or >1.3 mmol/L in females; Did not achieve target means the levels of HDL-C ≤ 1.0 mmol/L in males or ≤1.3 mmol/L in females.  ^a, b, c^ Each different subscript letter denotes a subset of age categories whose column proportions differ significantly from each other at *P* < 0.05. | | | | | | | | | | | |
|  | | | | | | | | | | | |
|  | | | | | | | | | | | |
